# The “parallel double deprivation”: how functional digital divides and gendered time constraints shape physical exercise participation in China

**DOI:** 10.3389/fpubh.2026.1841526

**Published:** 2026-07-08

**Authors:** Wangjie Li, Mingxian Wang, Jinjiang Li, Xiangwei Zhao

**Affiliations:** 1 Nanyang Normal University, Nanyang, China; 2 Nanyang Vocational College of Agriculture, Nanyang, China

**Keywords:** China family panel studies, gender inequality, health behavior, physical exercise, second-level digital divide, time displacement

## Abstract

**Background:**

Rapid societal digitalization is reshaping health behaviors, yet the assumption that internet connectivity uniformly promotes physical activity overlooks the functional heterogeneity of internet use commonly described as the second-level digital divide. Furthermore, the time-related implications of digital engagement may vary across social groups, particularly where gendered inequalities in unpaid domestic labor persist. This study examines how different forms of digital engagement are associated with physical exercise participation in China and explores whether these associations are stratified by gender and place of residence.

**Methods:**

Using nationally representative data from the 2022 China Family Panel Studies (CFPS) (*N* = 14,615), this study employed Probit, Ordered Logit, and Ordinary Least Squares (OLS) models to examine the associations between digital access, five functional dimensions of internet use—learning, socializing, consumption, entertainment, and work—and physical exercise outcomes. Oster’s bounding method was used to assess the sensitivity of key estimates to potential unobserved selection. Gender- and residence-stratified analyses were conducted to examine heterogeneity.

**Results:**

General internet access was positively associated with physical exercise. Among internet users, however, different digital functions showed divergent associations. Digital learning was positively associated with exercise participation, whereas digital work was negatively associated with participation after other digital functions were jointly considered. Gender-stratified analyses indicated that the negative association between digital work and exercise was more pronounced among women. Among women, unpaid housework and digital work were both negatively associated with exercise participation, suggesting a pattern consistent with what we conceptualize as “Parallel Double Deprivation.” In addition, digital entertainment was positively associated with exercise participation primarily among rural residents, indicating a possible spatially differentiated digital dividend.

**Conclusion:**

The relationship between digital engagement and physical exercise depends not only on internet access but also on the functions for which digital technologies are used. The findings suggest that work-related digital engagement and unpaid domestic labor may jointly constrain women’s health-promoting leisure time. Public health interventions should therefore pay closer attention to functional digital inequalities, gendered time constraints, and rural–urban differences in digital health opportunities.

## 1 Introduction

The rapid diffusion of digital technologies has reshaped the organization of everyday life and has generated important questions for public health (1, 2). On the one hand, internet access can expand opportunities for obtaining health information, accessing exercise-related resources, and participating in digitally mediated health communities (3, 4). On the other hand, the global increase in physical inactivity remains a major public health concern and a leading risk factor for non-communicable diseases (5, 6). As digital devices occupy an increasingly central place in work, leisure, and social interaction (7–9), an important empirical question arises: how is digital engagement related to physical exercise participation (10–12)?

Traditional public health and sociological literature has approached this question through the lens of the first-level digital divide, focusing primarily on whether individuals have internet access (13–15). Although this perspective remains important, it treats internet use as a relatively homogeneous condition and may therefore obscure substantial variation in how digital technologies are used. The internet is not only a channel for information acquisition but also a medium through which individuals allocate attention, leisure, work, and social time within a limited daily time budget (8, 9, 16, 17). For this reason, it is necessary to move beyond the binary distinction between users and non-users and examine the second-level digital divide: differences in the purposes, skills, and functional domains of internet use (13–15).

Different forms of digital engagement may have distinct implications for physical exercise. Learning-oriented internet use may be positively associated with exercise by improving access to health knowledge, increasing self-efficacy, and exposing individuals to fitness-related resources (3, 4, 18, 19), By contrast, work-related digital engagement may be associated with lower exercise participation if it extends work demands into non-work time and reduces opportunities for leisure-based physical activity (9, 20–22). Entertainment, socializing, and consumption may also show heterogeneous associations depending on whether they encourage sedentary screen time, provide social motivation, or connect users to offline activities (10, 20, 23, 24). These distinctions suggest that the health implications of digitalization cannot be adequately understood without disaggregating internet use into functional domains.

A further limitation in existing research is the tendency to treat the consequences of digital engagement as socially neutral, despite growing evidence that digital inequalities are structured by social class, gender, and other dimensions of social stratification (14, 25, 26). Time constraints associated with digital work or screen-based activities may not affect all groups equally. In particular, gender remains a key axis of inequality in the allocation of unpaid domestic labor (27–30). Women often face greater responsibility for household tasks and caregiving, and these obligations may limit their discretionary time for physical exercise (31–33). At the same time, the boundary-blurring character of work-related digital engagement may further intensify time pressure, especially when professional availability extends beyond formal working hours (7, 9, 21). Prior research has examined gender inequalities in digital work and unpaid labor, but less attention has been paid to how these two forms of time constraint are jointly associated with health-promoting leisure behaviors such as physical exercise (28, 31–34).

To address this gap, this study proposes “Parallel Double Deprivation” as an interpretive framework for understanding how work-related digital engagement and unpaid domestic labor may operate as parallel constraints on women’s exercise participation. Drawing on nationally representative data from the 2022 China Family Panel Studies (35), this study has three objectives. First, it examines how digital access and different functional dimensions of internet use are associated with physical exercise participation, frequency, and duration. Second, it investigates whether the association between digital work and exercise differs by gender and whether unpaid housework provides additional evidence for a pattern consistent with Parallel Double Deprivation. Third, it explores whether the association between digital entertainment and exercise varies across rural and urban residents. By shifting attention from digital access to functional digital engagement, this study contributes to a more differentiated understanding of digital inequality, gendered time constraints, and physical exercise participation in contemporary China.

## 2 Methodology

### 2.1 Data source and sample selection

The empirical analysis draws on data from the China Family Panel Studies (CFPS), a nationally representative, longitudinal social survey conducted by the Institute of Social Science Survey (ISSS) at Peking University. The CFPS employs a multistage, implicit stratification, probability proportional to size (PPS) sampling design, covering 25 provinces/municipalities/autonomous regions in China (35).

This study uses the 2022 CFPS wave. To ensure data integrity, particularly for key demographic variables such as years of schooling, we retroactively supplemented missing data using information from the 2020 wave. The sample selection proceeded as follows. First, we restricted the sample to working-age adult aged 18 to 65. Second, we excluded observations with severe logical inconsistencies or extensive missing values on core variables. To rigorously address the remaining item non-response (e.g., occasional missing values in control variables) and avoid the statistical bias inherent in list-wise deletion, we employed the Multiple Imputation (MI) with 5 imputations, following standard methodological guidelines for complex survey designs (36). Our final analytical sample comprises 14,615 valid observations. To account for the complex survey design and ensure the population-level representativeness, all descriptive and regression analyses incorporated individual-level sampling weights (*p*weight) and clustered robust standard errors at the community/village level.

### 2.2 Measures

#### 2.2.1 Dependent variable: physical exercise

Physical exercise behavior is operationalized across three complementary dimensions: participation, frequency, and duration. Consistent with the CFPS questionnaire guidelines and the World Health Organization’s recommendations on physical activity for health (37), physical exercise is strictly defined as indoor or outdoor activities aimed at strengthening the body and relaxing the mind, explicitly excluding commuting activities (e.g., walking or cycling to work).

*Participation*: A binary indicator of whether the respondent engaged in physical exercise in the past 12 months. It is coded as 1 if the self-reported exercise frequency is greater than zero, and 0 if the respondent reported “never participating.”

*Frequency*: An ordinal variable capturing the regularity of exercise in the past 12 months, ranging from 0 (“never”) to 7 (“twice or more per day”).

*Duration*: Measured as the average time spent per exercise session (in minutes). To address positive skewness and retain observations with zero duration, we applied a logarithmic transformation, denoted as ln (duration + 1).

#### 2.2.2 Independent variables: digital access and functional digital engagement

*Digital access (first-level divide)*: A dummy variable indicating internet use, coded as 1 for users and 0 for non-users.

*Digital functional dimensions (second-level divide)*: To capture functional digital engagement among internet users: digital learning, digital socializing, digital consumption, digital entertainment, and digital work. Recognizing that digital engagement encompasses both perceived importance and actual usage, each dimension aggregates the respondent’s cognitive perception of the internet’s importance in a specific domain (measured on a 5-point Likert scale) with their behavioral frequency (e.g., from non-use to daily use). To ensure commensurability, all raw indicators were min-max normalized to a [0, 1] continuous scale before being averaged into the final indices:

Digital learning (D1) combines the perceived importance of the internet for learning with the frequency of online learning behavior. This indicator captures both the subjective relevance of the internet for learning and actual engagement in online learning activities.

Digital socializing (D2) combines the perceived importance of the internet for maintaining social relationships with the frequency of posting on WeChat Moments. It captures the extent to which respondents use digital platforms for social interaction and relationship maintenance.

Digital consumption (D3) combines the perceived importance of the internet for daily life with the frequency of online shopping. It reflects the role of the internet in consumption-related and everyday-life activities.

Digital entertainment (D4) is based on the frequency of entertainment-oriented digital behaviors, specifically short-video viewing and online gaming. Because the available CFPS items for this domain mainly capture behavioral frequency rather than perceived importance, this measure should be interpreted as a behavior-based indicator of digital entertainment engagement.

Digital work (D5) is measured by the perceived importance of the internet for professional work, normalized to the 0–1 range. This variable captures the respondent’s subjective assessment of the internet’s relevance to work-related activities. It should therefore be interpreted as perceived work-related digital reliance rather than a direct measure of time spent working online.

Because the five indicators draw on different types of CFPS items, they should be understood as domain-specific measures of functional digital engagement rather than fully equivalent psychometric scales. Accordingly, we do not treat them as interchangeable latent constructs. Instead, we use them to distinguish substantively different forms of internet use and compare their adjusted associations with physical exercise outcomes.

To assess whether the joint inclusion of the five digital dimensions raised multicollinearity concerns, we calculated variance inflation factors using an auxiliary OLS regression with the same set of predictors as the joint Probit model. As shown in Supplementary Table S1, the mean VIF was 1.36 and the maximum VIF was 1.97. The VIF values for the five digital dimensions ranged from 1.24 to 1.47, indicating that multicollinearity was not a serious concern.

In the empirical analysis, the five indicators were first examined separately and then entered jointly to assess their adjusted associations with physical exercise outcomes after accounting for other forms of digital engagement.

#### 2.2.3 Time-constraint and moderating variables

To examine whether gendered time constraints are associated with physical exercise participation, this study included unpaid housework as an additional explanatory variable.

*Unpaid housework*: Measured as the self-reported average hours per day spent on domestic chores (excluding time spent caring for family members). For respondents with missing daily averages, we imputed the value using a weighted average of their reported weekday and weekend housework hours (Weekday × 5 + Weekend × 2)/7.

*Moderators*: Gender (1 = Male, 0 = Female) and Hukou registration status (1 = Urban, 0 = Rural).

#### 2.2.4 Control variables

To minimize omitted variable bias, we controlled for a comprehensive set of covariates, including: age (continuous), gender, marital status (partnered = 1), years of education (continuous), household income (log-transformed), subjective social status (scale of 1 to 5), self-rated health, presence of chronic diseases (1 = yes), Hukou, and regional dummies (Eastern, Central, Western).

### 2.3 Empirical strategy

Given the distinct distributions of our dependent variables, we adopted a suite of econometric models to estimate the effects of digital engagement on physical exercise. Because the CFPS 2022 data are cross-sectional, the estimated coefficients are interpreted as conditional associations rather than causal effects.

First, to estimate the impact on exercise participation (a binary outcome), we used a Probit model. The baseline specification is as shown in Equation 1:


$$\mathrm{Pr}({\text{Exercise}}_{i}=1|X_{i})=\Phi (\begin{array}{l} \alpha_{0}+\beta_{1}\;\text{Digital}\_{\text{Access}}_{i}\\ +\gamma X_{i}+\mu_{c}+\varepsilon_{i} \end{array})$$ (1)

For the mechanism analysis examining functional heterogeneity among internet users, the model is as shown in Equation 2:


$$\mathrm{Pr}({\text{Exercise}}_{i}=1|X_{i})=\Phi (\alpha_{0}+\sum_{k=1}^{5}\beta_{k}D_{\mathrm{ki}}+\gamma X_{i}+\mu_{c}+\varepsilon_{i})$$ (2)

where Φ(.) represents the cumulative distribution function of the standard normal distribution. $D_{\mathrm{ki}}$ represents the five functional dimensions of digital usage (learning, socializing, consumption, entertainment, work) for individual *i*. $X_{i}$ is the vector of control variables. $\mu_{c}$ denotes the community-level fixed effects (handled via clustered standard errors), and $\varepsilon_{i}$ is the random error term.

Second, for exercise frequency (an ordinal categorical variable ranging from 0 to 7), we employed an Ordered Logit (Ologit) model.

Third, for exercise duration (a continuous variable), we applied an Ordinary Least Squares (OLS) regression as shown in Equation 3:


$$\ln({\text{Duration}}_{i}+1)=\alpha_{0}+\sum_{k=1}^{5}\beta_{k}D_{\mathrm{ki}}+\gamma X_{i}+\mu_{c}+\varepsilon_{i}$$ (3)

Finally, to rigorously test the “Parallel Double Deprivation” hypothesis, we conducted group-specific regressions by gender and region and introduced interaction terms (e.g., Digital_work×Gender ) to formally test for moderating effects.

Furthermore, a persistent concern in observational studies is the potential for omitted variable bias (e.g., unobservable personality traits affecting both digital habits and exercise). To rigorously address this, we implemented Oster’s (38) bounding approach. This method evaluates coefficient stability and *R*^2^ movements after including covariates to assess how large the unobservable selection bias would need to be to drive the true treatment effect to zero. Following Oster’s methodological recommendations, we set the maximum possible *R*^2^ (*R*_max_) at 1.3 times the *R*^2^ of the fully controlled model, and calculated both the identified bounding set (assuming proportional selection, *δ* = 1) and the critical delta (δ*).

## 3 Results

### 3.1 Descriptive statistics

Table 1 presents the descriptive statistics for the key variables based on the final analytic sample of 14,615 respondents. The statistics reveal the landscape of physical (in)activity and digital engagement in contemporary China.

**Table 1:** Descriptive statistics for key variables.

Continuous variables	Meaning	Values	Mean ± SD	Min	Max
Exercise frequency	Frequency of physical exercise participation	0 (never) to 7 (twice daily)	1.705 ± 2.280	0.00	7.00
Ln_Exercise duration	Log-transformed minutes per exercise session	Log of minutes per session	1.624 ± 1.928	0.00	5.60
Exercise participation	Participation in physical exercise (dichotomous)	0 (never), 1 (any)	0.425 ± 0.494	0.00	1.00
Digital access	Internet usage status	0 (no), 1 (yes)	0.784 ± 0.412	0.00	1.00
Digital learning	Composite index of learning cognition and behavior	0 to 1	0.401 ± 0.263	0.00	1.00
Digital socializing	Composite index of social importance and Moments usage	0 to 1	0.509 ± 0.231	0.00	1.00
Digital consumption	Composite index of daily life cognition and online shopping	0 to 1	0.450 ± 0.269	0.00	1.00
Digital entertainment	Composite index of entertainment cognition and short video/gaming	0 to 1	0.485 ± 0.260	0.00	1.00
Digital work	Cognitive indicator of work-related internet importance	0 to 1	0.626 ± 0.370	0.00	1.00
Gender	Respondent’s gender	0 (female), 1 (male)	0.512 ± 0.500	0.00	1.00
Age	Respondent’s age in years	Years	42.810 ± 13.381	18.00	65.00
partnered	Marital status (having a spouse/partner)	0 (no), 1 (yes)	0.753 ± 0.431	0.00	1.00
Years of education	Completed years of formal education	Years of formal education	10.697 ± 5.534	0.00	23.00
Urban	Household registration (Hukou) type	0 (rural), 1 (urban)	0.282 ± 0.450	0.00	1.00
Ln_ household income	Natural log of per capita household income (CNY)	Log of per capita household income	10.056 ± 1.076	0.00	15.75
Subjective social status	Self-placed local social status	1 (very low) to 5 (very high)	2.955 ± 1.037	1.00	5.00
Self-rated health	Overall self-rated health	1 (worst) to 5 (best)	3.182 ± 1.149	1.00	5.00
Chronic disease	Chronic disease diagnosis in the past 6 months	0 (no), 1 (yes)	0.137 ± 0.344	0.00	1.00

Categorical variable	Meaning	Category	*N*	%
Region	Geographic region	West	4,208	28.79
		Central	4,352	29.78
		East	6,055	41.43

Continuous variables are reported as mean ± SD, together with Min, and Max. Binary variables are reported as mean ± SD where the mean equals the proportion. All statistics are based on the analytic sample (*N* = 14,615).

Overall participation in physical exercise was relatively limited. Approximately 42.5% of respondents reported engaging in physical exercise during the past 12 months. The mean exercise frequency score was 1.70 on the original 0–7 scale, indicating generally low levels of regular exercise in the sample.

Regarding digital engagement, 78.4% of respondents reported internet access, indicating a high level of general connectivity while also reflecting remaining first-level digital inequality. Among internet users, the five functional dimensions of digital engagement displayed different average levels. Digital work had the highest mean score (*M* = 0.626, SD = 0.370), suggesting that work-related reliance on the internet was relatively prominent in this sample. By contrast, digital learning had the lowest mean score (*M* = 0.401, SD = 0.263). The other dimensions showed moderate levels of engagement: digital socializing (*M* = 0.509, SD = 0.231), digital entertainment (*M* = 0.485, SD = 0.260), and digital consumption (*M* = 0.450, SD = 0.269). These descriptive patterns suggest heterogeneity in how respondents used the internet across functional domains, which motivates the subsequent multivariate analysis.

The sociodemographic profile of the sample indicates an average age of 42.8 years (SD = 13.4), with a balanced gender distribution (51.2% male). The majority of respondents were partnered (75.3%). Average educational attainment was approximately 10.7 years, roughly equivalent to a high school education. Regarding spatial and socioeconomic stratification, 28.2% of the sample held an urban Hukou (household registration), and the sample was distributed across the Eastern (41.4%), Central (29.8%), and Western (28.8%) regions of China, supporting broad national coverage. Health-wise, respondents reported moderate levels of subjective social status (*M* = 2.96, on a 1–5 scale) and self-rated health (*M* = 3.18, on a 1–5 scale), with 13.7% having been diagnosed with a chronic disease in the past 6 months.

### 3.2 Bivariate analysis by digital access status

Table 2 presents the bivariate comparisons of physical exercise, digital engagement, and sociodemographic characteristics between respondents without internet access (*n* = 3,164) and those with internet access (*n* = 11,451). Independent-sample t-tests and chi-square tests indicate statistically significant differences between the two groups across all observed variables (*p* < 0.01), suggesting that internet access is closely associated with broader demographic, socioeconomic, and health-related disparities.

**Table 2:** Bivariate comparisons by digital access status.

Variable	No access (*n* = 3,164)	Access (*n* = 11,451)	Mean diff	*p*-value
Continuous variables	Mean ± SD	Mean ± SD		
Exercise frequency	1.160 ± 2.239	1.855 ± 2.269	−0.695	<0.01
Ln_exercise duration	0.899 ± 1.640	1.824 ± 1.953	−0.925	<0.01
Digital learning	0.192 ± 0.204	0.458 ± 0.247	−0.267	<0.01
Digital socializing	0.311 ± 0.277	0.564 ± 0.182	−0.253	<0.01
Digital consumption	0.184 ± 0.204	0.523 ± 0.237	−0.339	<0.01
Digital entertainment	0.178 ± 0.192	0.569 ± 0.208	−0.391	<0.01
Digital work	0.383 ± 0.412	0.693 ± 0.328	−0.309	<0.01
Age	53.917 ± 8.408	39.741 ± 12.864	14.175	<0.01
Years of education	7.458 ± 6.294	11.592 ± 4.944	−4.134	<0.01
Ln_household income	9.618 ± 1.123	10.177 ± 1.030	−0.559	<0.01
Subjective social status	3.305 ± 1.174	2.859 ± 0.974	0.447	<0.01
Self-rated health	2.933 ± 1.354	3.251 ± 1.075	−0.318	<0.01
Categorical variables	*n* (%)	*n* (%)	*χ* ^2^	
Exercise participation	751 (23.7%)	5,466 (47.7%)	584.082	<0.01
Gender (male)	1,476 (46.6%)	6,006 (52.4%)	33.374	<0.01
Partnered (yes)	2,832(89.5%)	8,177(71.4%)	436.901	<0.01
Chronic disease (yes)	634 (20.0%)	1,364 (11.9%)	138.712	<0.01
Urban (yes)	484(15.3%)	3,631(31.7%)	330.095	<0.01
Region			125.852	<0.01
West	1,109 (35.1%)	3,099 (27.1%)		
Central	1,004 (31.7%)	3,348 (29.2%)		
East	1,051 (33.2%)	5,004 (43.7%)		

*p*-values are derived from independent sample *t*-tests for continuous variables and Chi-square tests for binary/categorical variables.

Respondents with internet access reported higher levels of physical exercise than those without access. The exercise participation rate was 47.7% among internet users, compared with 23.7% among non-users. Internet users also reported higher exercise frequency scores (1.86 vs. 1.16 on the original 0–7 scale) and longer log-transformed exercise duration. These descriptive differences are consistent with the possibility of a digital health advantage, although they should not be interpreted as causal evidence.

The two groups also differed substantially in their sociodemographic profiles. Compared with respondents without internet access, internet users were younger on average (39.7 vs. 53.9 years), had more years of education (11.6 vs. 7.5 years), and reported higher household income. They also reported better self-rated health and a lower prevalence of chronic disease (11.9% vs. 20.0%). These differences indicate that digital access is strongly patterned by age, education, income, and health status.

Taken together, the bivariate results suggest that the observed association between internet access and physical exercise may partly reflect compositional differences between connected and unconnected respondents. Therefore, unadjusted comparisons are insufficient for assessing the independent association between digital engagement and exercise behavior. The following multivariate analyses adjust for a range of sociodemographic, socioeconomic, and health-related covariates to examine whether these associations persist after accounting for observed differences between groups.

### 3.3 Baseline regression: the first-level digital divide and physical exercise

To examine whether the descriptive differences observed in Table 2 persist after adjusting for observed covariates, we estimated multivariate baseline regressions. As shown in Table 3, we used a Probit model for exercise participation (Model 1), an Ordered Logit model for exercise frequency (Model 2), and an Ordinary Least Squares (OLS) model for log-transformed exercise duration (Model 3).

**Table 3:** Baseline regression: digital access and physical exercise.

Variable	(1) Participation	(2) Frequency	(3) Ln (duration)
	Probit	Ordered logit	OLS
Digital access	0.381***	0.704***	0.491***
	(0.049)	(0.094)	(0.060)
Gender (male)	0.035	0.008	0.054
	(0.025)	(0.039)	(0.035)
Age	−0.007***	0.007**	−0.009***
	(0.002)	(0.003)	(0.002)
Partnered (yes)	−0.286***	−0.449***	−0.496***
	(0.041)	(0.056)	(0.056)
Years of education	0.030***	0.040***	0.040***
	(0.003)	(0.005)	(0.004)
Ln_household income	0.101***	0.125***	0.139***
	(0.031)	(0.041)	(0.036)
Subjective social status	0.070***	0.088***	0.088***
	(0.021)	(0.029)	(0.024)
Self-rated health	0.037***	0.096***	0.057***
	(0.013)	(0.023)	(0.017)
Chronic disease (yes)	0.247***	0.442***	0.375***
	(0.044)	(0.075)	(0.061)
Urban (yes)	0.515***	0.732***	0.774***
	(0.041)	(0.049)	(0.050)
Central	0.009	−0.011	−0.014
	(0.046)	(0.064)	(0.058)
East	−0.034	−0.117**	−0.062
	(0.041)	(0.059)	(0.053)
Constant/Intercept	−1.816***	—	−0.523*
	(0.240)		(0.279)
Cut points	—	3.303***, 3.426***, 3.727***, 4.414***, 4.826***, 4.921***, 7.024***	—
*N*	14,615	14,615	14,615

Unstandardized coefficients are presented with robust standard errors clustered at the community/village level in parentheses. The reference category for region is the Eastern region. For the Ordered Logit model, seven cut points are reported; standard errors for cut points are omitted for brevity but are available upon request. *p < 0.1, **p < 0.05, ***p < 0.01.

The results show a consistently positive association between digital access and physical exercise across all three outcomes. After adjusting for sociodemographic, socioeconomic, and health-related covariates, digital access remains positively and significantly associated with exercise participation (*β* = 0.381, *p* < 0.01), exercise frequency (*β* = 0.704, *p* < 0.01), and exercise duration (*β* = 0.491, *p* < 0.01). These findings suggest that individuals with internet access are more likely to report higher levels of physical exercise than those without access. However, given the cross-sectional nature of the data, these associations should not be interpreted as evidence that internet access directly causes higher exercise participation.

The control variables also show patterns broadly consistent with previous research on physical activity. Education, household income, and urban hukou status are positively associated with physical exercise across the three models, indicating that human capital, economic resources, and urban residence are linked to higher levels of exercise engagement. Being partnered is negatively associated with exercise participation (*β* = −0.286, *p* < 0.01 in Model 1), which may reflect competing time demands related to family and household responsibilities. By contrast, having a diagnosed chronic disease is positively associated with exercise participation (*β* = 0.247, *p* < 0.01 in Model 1), possibly suggesting that individuals with health conditions may be more attentive to health-related behaviors.

Although these baseline models indicate a positive association between general internet access and physical exercise, they treat digital engagement as a binary condition. This approach cannot capture differences in how the internet is used. Different forms of digital engagement may have distinct associations with physical exercise, depending on whether they are related to learning, social interaction, consumption, entertainment, or work. Therefore, the following analysis focuses on internet users and examines the functional heterogeneity of digital engagement.

### 3.4 Functional heterogeneity: the second-level digital divide and physical exercise

While the baseline models indicate a positive association between general internet access and physical exercise, treating internet use as a single binary condition may obscure variation across different forms of digital engagement. To examine this functional heterogeneity, we restricted the sample to internet users and estimated the associations between the five digital dimensions and exercise participation (Table 4).

**Table 4:** Individual and joint effects of digital functions on exercise participation.

Variable	(1)	(2)	(3)	(4)	(5)	(6)
Digital learning	0.926***					0.957***
	(0.048)					(0.058)
Digital socializing		0.408***				0.021
		(0.052)				(0.065)
Digital consumption			0.376***			−0.042
			(0.047)			(0.059)
Digital entertainment				0.426***		0.168***
				(0.048)		(0.059)
Digital work					0.231***	−0.114***
					(0.032)	(0.040)
Controls	Yes	Yes	Yes	Yes	Yes	Yes
Regional effects	Yes	Yes	Yes	Yes	Yes	Yes
Constant	−1.985***	−1.894***	−1.819***	−1.898***	−1.791***	−2.029***
	(0.140)	(0.144)	(0.140)	(0.142)	(0.142)	(0.142)
*N*	14,615	14,615	14,615	14,615	14,615	14,615

Unstandardized coefficients from Probit regressions are reported with robust standard errors in parentheses. The dependent variable is exercise participation (binary). All models control for gender, age, partnered status, years of education, household income (ln), subjective social status, self-rated health, chronic disease, and urban Hukou. *p < 0.1, **p < 0.05, ***p < 0.01.

Models 1 through 5 report the results when each digital dimension is entered separately. In these models, digital learning, digital socializing, digital consumption, digital entertainment, and digital work are all positively and significantly associated with exercise participation (*p* < 0.01). These results suggest that, when considered individually, each dimension of digital engagement is correlated with a higher likelihood of reporting physical exercise.

However, the five dimensions are not mutually exclusive, and respondents may engage in several forms of digital activity simultaneously. To assess the association of each dimension while accounting for the others, Model 6 includes all five indicators in the same specification. The results show a more differentiated pattern. Digital learning remains positively and significantly associated with exercise participation (*β* = 0.957, *p* < 0.01), whereas digital work becomes negatively associated with exercise participation (*β* = −0.114, *p* < 0.01). Digital entertainment remains positively associated, although its coefficient is smaller in magnitude than that of digital learning. These results indicate that the observed associations differ across functional forms of internet use and that the direction of the association for digital work depends on model specification.

**Figure 1:**
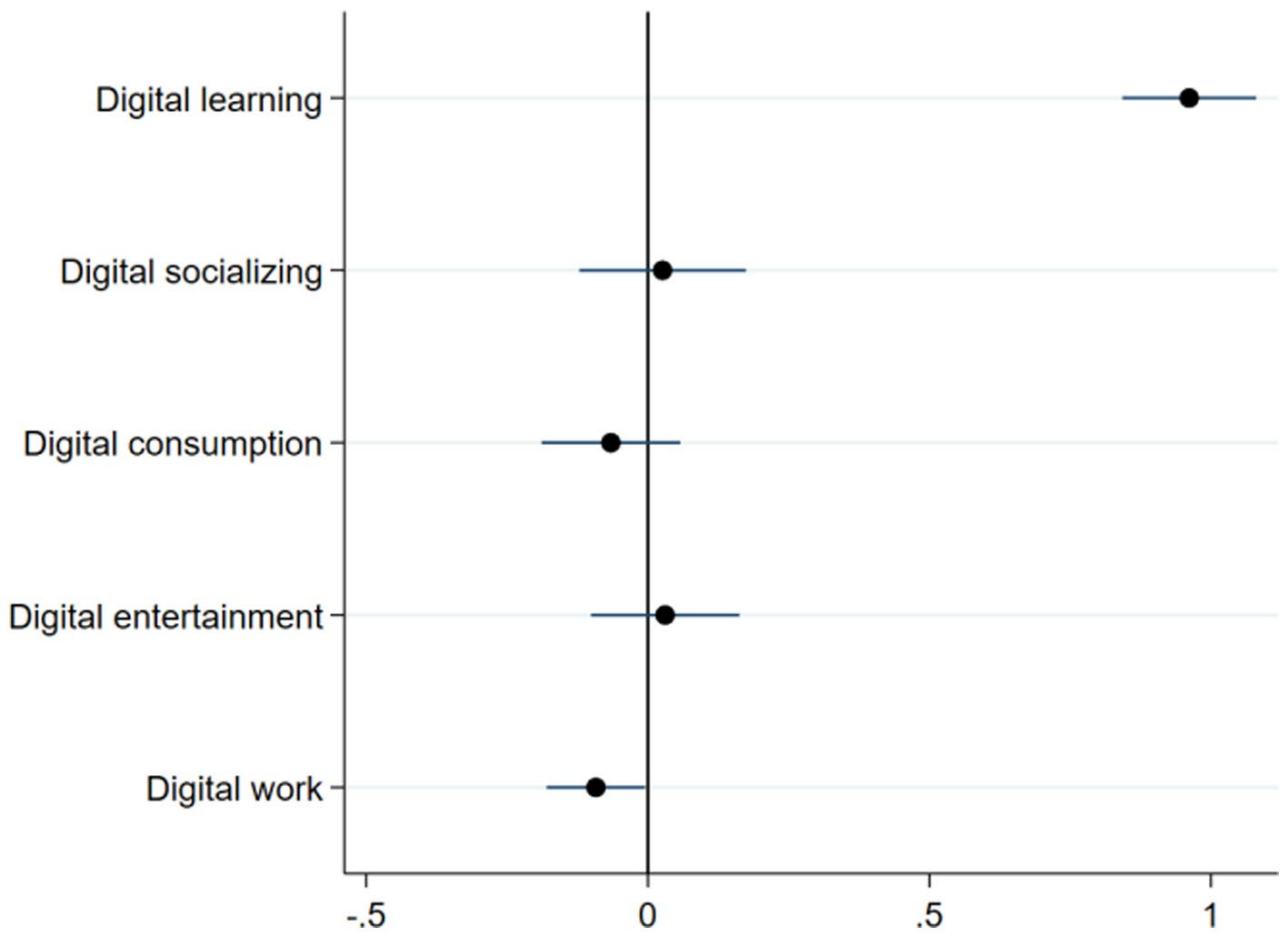
Coefficient plot: effects of digital functions on exercise participation. The horizontal bars represent 95% confidence intervals. The vertical line indicates a coefficient of zero.

Figure 1 summarizes these coefficient estimates visually. The contrast between the separate and joint models suggests that different forms of digital engagement may be linked to exercise participation in different ways. In particular, the negative coefficient for digital work in the joint model is consistent with the possibility that work-related digital engagement is associated with constraints on leisure time, although this interpretation should be treated as suggestive rather than conclusive.

**Figure 2:**
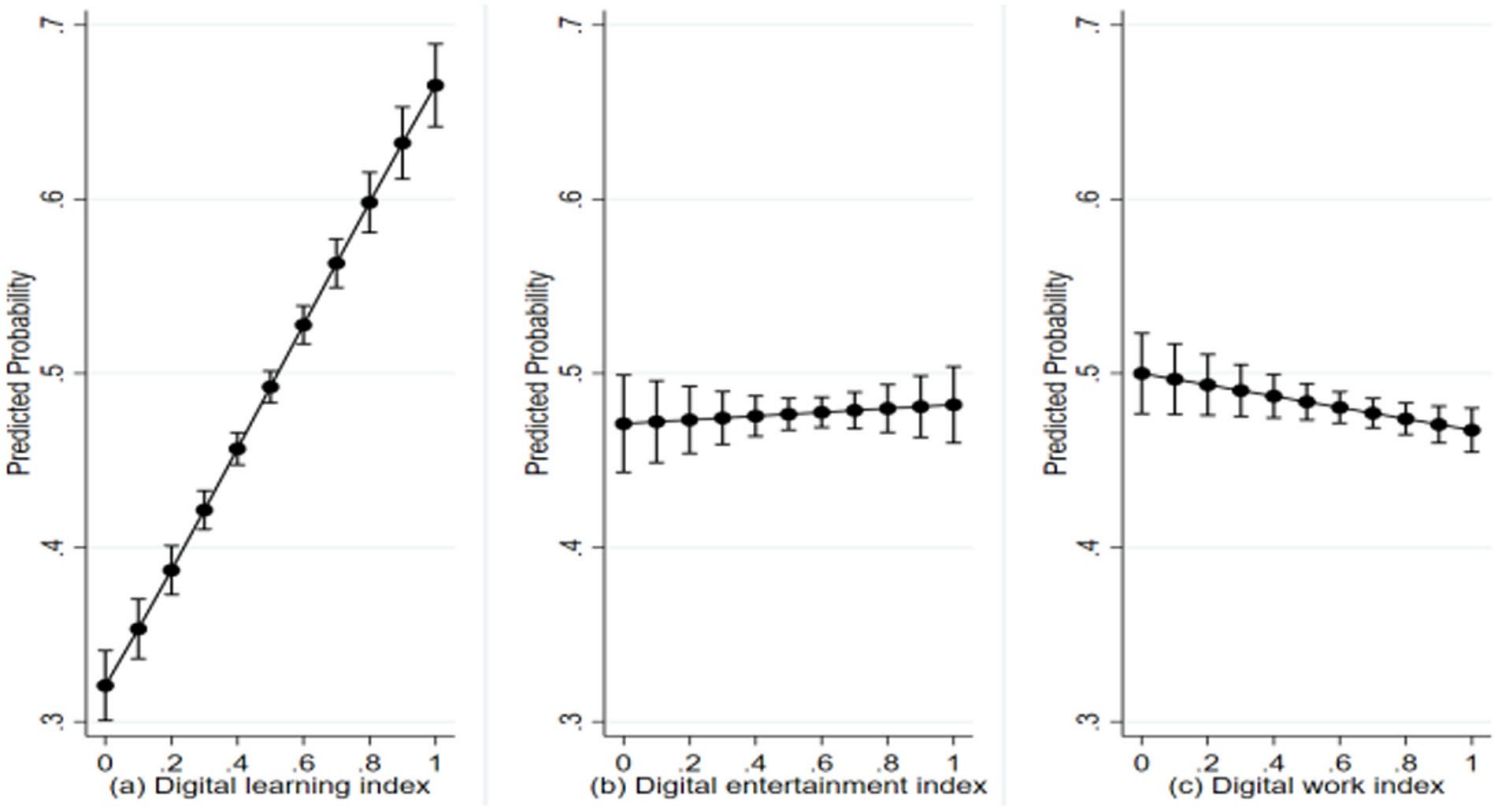
Predicted probabilities: divergent effects of digital engagement on exercise. **(a)** Digital learning; **(b)** Digital entertainment; **(c)** Digital work.

To further illustrate the substantive magnitude of these associations, Figure 2 presents predicted probabilities of exercise participation across the observed range of digital learning, digital entertainment, and digital work. Figure 2a shows that higher levels of digital learning are associated with a higher predicted probability of exercise participation, increasing from approximately 0.30 to 0.70 across the index range. Figure 2b shows a more modest positive gradient for digital entertainment. By contrast, Figure 2c indicates a negative gradient for digital work, with the predicted probability of exercise participation declining from approximately 0.55 to 0.45 across the index range. These predicted probabilities help illustrate that the association for digital work is statistically significant but substantively more modest than the association observed for digital learning.

Overall, the results suggest that the relationship between digital engagement and physical exercise is not uniform across different forms of internet use. Digital learning is positively associated with exercise participation, whereas digital work is negatively associated with it in the fully adjusted joint model. This pattern is broadly consistent with the view that different digital practices may be linked to health behavior through different pathways. The next section examines whether these associations vary across gender and hukou groups.

### 3.5 Heterogeneity analysis by gender and hukou status

**Figure 3:**
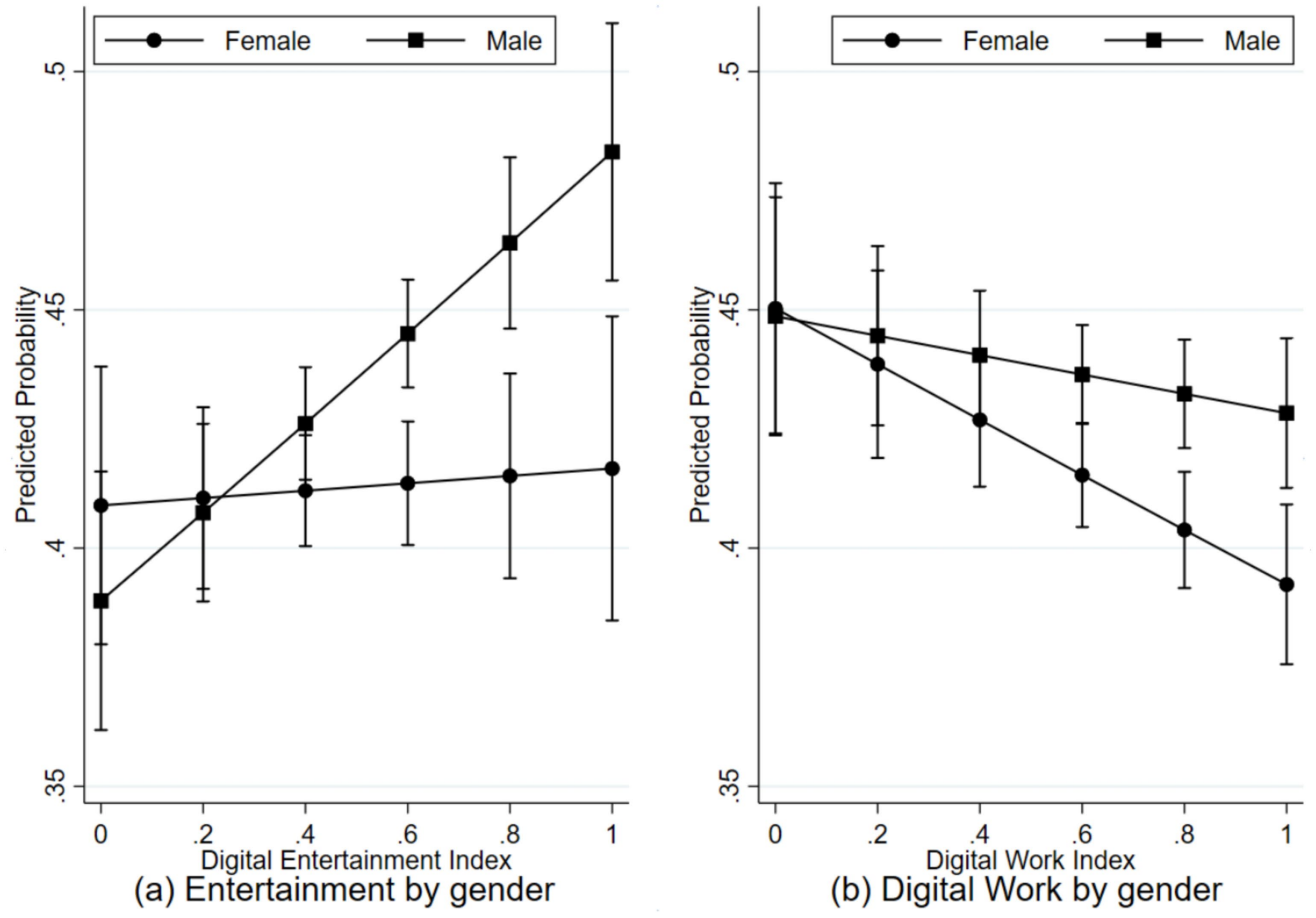
Gender asymmetry in the effects of digital work and entertainment. **(a)** Predictive margins of digital entertainment by gender. **(b)** Predictive margins of digital work by gender, with 95% confidence intervals.

**Figure 4:**
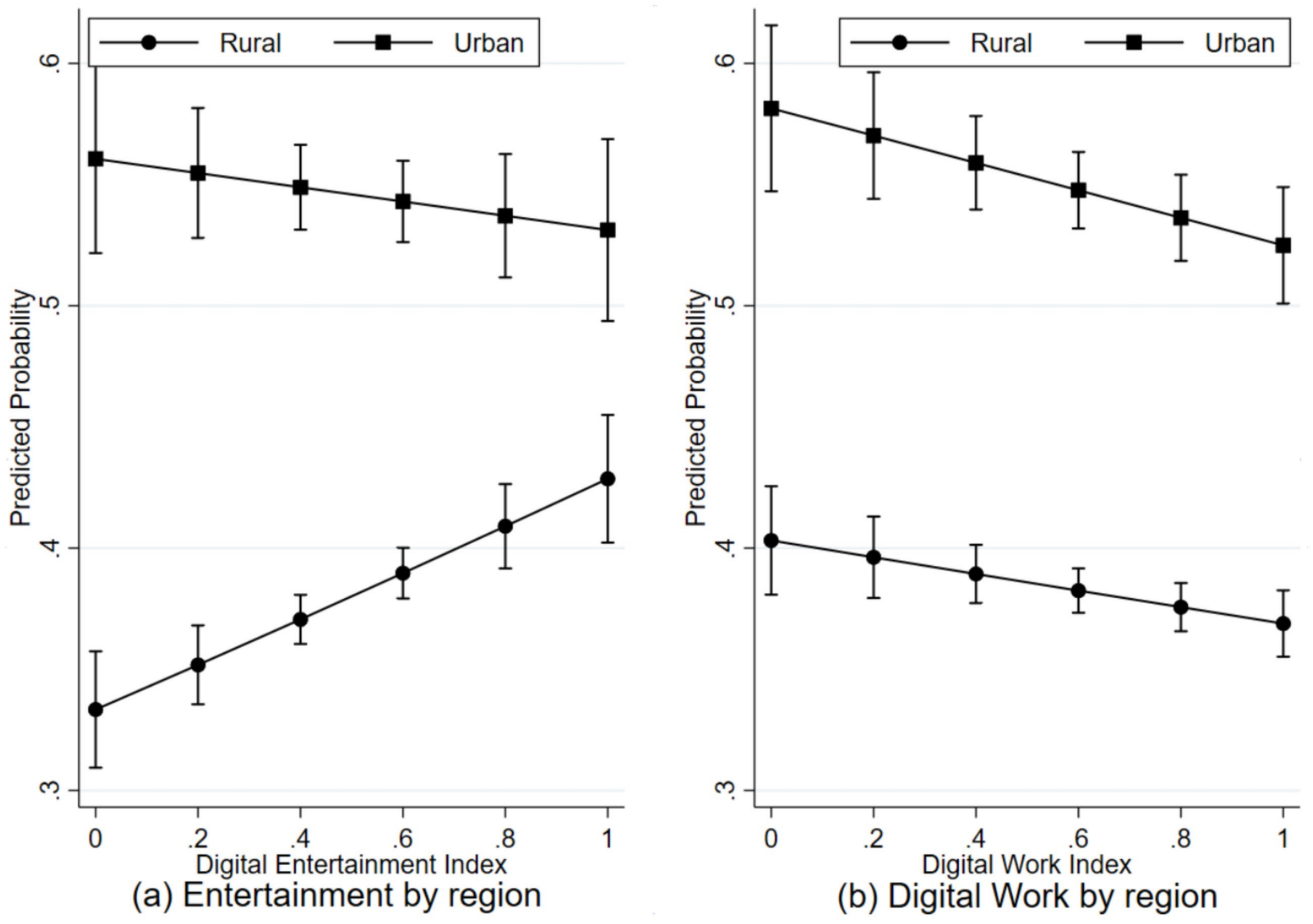
Urban–rural divergence: spatial heterogeneity in digital effects. **(a)** Predictive margins of digital entertainment by Hukou status. **(b)** Predictive margins of digital work by Hukou status, with 95% confidence intervals.

The joint model in Table 4 indicates that different forms of digital engagement are associated with exercise participation in different ways. To examine whether these associations vary across social groups, we conducted stratified analyses by gender and hukou status, as reported in Table 5. The corresponding predicted probabilities are presented in Figures 3 and 4. These analyses are intended to describe heterogeneity in associations rather than to establish group-specific causal mechanisms.

**Table 5:** Heterogeneity analysis: digital engagement and exercise participation by gender and hukou.

Variable	(1) Male	(2) Female	(3) Rural	(4) Urban
Digital learning	1.054***	0.853***	0.992***	0.836***
	(0.080)	(0.085)	(0.070)	(0.103)
Digital socializing	−0.019	0.144	−0.006	0.042
	(0.091)	(0.093)	(0.078)	(0.118)
Digital consumption	−0.002	0.000	−0.015	−0.091
	(0.082)	(0.086)	(0.071)	(0.107)
Digital entertainment	0.134*	0.114	0.219***	0.039
	(0.081)	(0.089)	(0.071)	(0.109)
Digital work	−0.089	−0.136**	−0.110**	−0.121
	(0.057)	(0.058)	(0.048)	(0.077)
Controls	Yes	Yes	Yes	Yes
Regional effects	Yes	Yes	Yes	Yes
Constant	−1.917***	−2.293***	−1.686***	−2.760***
	(0.221)	(0.198)	(0.171)	(0.287)
*N*	7,482	7,133	10,500	4,115

Unstandardized coefficients from Probit regressions are reported with robust standard errors in parentheses. The dependent variable is exercise participation (binary). All models control for age, partnered status, years of education, household income (ln), subjective social status, self-rated health, and chronic disease. Models 1-2 additionally control for Hukou; Models 3-4 additionally control for gender. *p < 0.1, **p < 0.05, ***p < 0.01.

The gender-stratified results suggest a possible gender difference in the association between digital work and exercise participation. Among female respondents, digital work is negatively and significantly associated with exercise participation (*β* = −0.136, *p* < 0.05). Among male respondents, the coefficient is also negative but not statistically significant (*β* = −0.089, *p* > 0.1). As shown in Figure 3b, the predicted probability of exercise participation declines more clearly among women as the digital work index increases, whereas the corresponding pattern among men is flatter. This pattern is consistent with the possibility that work-related digital engagement may be more closely linked to reduced exercise participation among women. However, this interpretation should remain cautious unless formally supported by a statistically significant interaction term.

Digital entertainment shows a different gender pattern. As shown in Table 5 and Figure 3a, digital entertainment is weakly and positively associated with exercise participation among men (*β* = 0.134, *p* < 0.1), whereas the association is not statistically significant among women. This result suggests that the positive association between entertainment-oriented digital use and exercise participation may be more evident among male respondents, although the relatively weak significance level warrants cautious interpretation.

The hukou-stratified results indicate spatial heterogeneity in the association between digital entertainment and exercise participation. Digital entertainment is positively and significantly associated with exercise participation among rural respondents (*β* = 0.219, *p* < 0.01), but the corresponding coefficient is not statistically significant among urban respondents (*β* = 0.039, *p* > 0.1). Figure 4a, illustrates this contrast: the predicted probability of exercise participation increases with digital entertainment among rural respondents, while the urban pattern is comparatively flat. One possible interpretation is that entertainment-oriented digital platforms may be more closely connected to offline physical activities in rural settings, where formal fitness infrastructure and organized leisure opportunities may be more limited. This interpretation remains suggestive and should not be read as direct evidence of a platform-driven exercise mechanism.

Digital learning remains positively and significantly associated with exercise participation across all gender and hukou subgroups (*p* < 0.01), suggesting that its positive association with exercise participation is relatively consistent across social groups. For digital work, as shown in Figure 4b, both rural and urban respondents show negative coefficients. The coefficient is statistically significant among rural respondents (*β* = −0.110, *p* < 0.05), whereas it is not significant among urban respondents despite a similar magnitude (*β* = −0.121, *p* > 0.1). Therefore, the evidence suggests a negative association between digital work and exercise participation in some subgroups, but it does not justify a strong claim that this pattern is uniformly present across all spatial contexts.

Overall, the heterogeneity analyses show that the associations between digital engagement and exercise participation differ by gender and hukou status. The negative association between digital work and exercise participation appears more pronounced among women, while the positive association between digital entertainment and exercise participation is more evident among rural respondents. These subgroup patterns provide a basis for the subsequent analysis of unpaid housework as a potential gendered constraint, but they should be interpreted as exploratory evidence rather than definitive proof of structural mechanisms.

### 3.6 Mechanism analysis: exploring “parallel double deprivation”

The heterogeneity analysis suggests that the negative association between digital work and exercise participation may be more pronounced among women. To further examine this gendered pattern, we introduced unpaid housework into the gender-stratified models, as reported in Table 6. Unpaid housework is used as an indicator of domestic time demands and traditional gendered labor allocation. The purpose of this analysis is to assess whether digital work and unpaid housework are independently associated with women’s exercise participation, rather than to establish a definitive causal mechanism.

**Table 6:** The “double deprivation” mechanism: grouped regressions by gender with unpaid housework.

Variable	(1) Male	(2) Male	(3) Female	(4) Female
	Baseline	+ Housework	Baseline	+ Housework
Digital learning	1.037***	1.037***	0.870***	0.869***
	(0.083)	(0.083)	(0.090)	(0.090)
Digital work	−0.009	−0.011	−0.177***	−0.181***
	(0.062)	(0.062)	(0.064)	(0.065)
Unpaid housework		0.008		−0.034***
		(0.012)		(0.012)
Controls	Yes	Yes	Yes	Yes
Regional effects	Yes	Yes	Yes	Yes
Constant	−1.963***	−1.975***	−2.037***	−1.966***
	(0.238)	(0.239)	(0.224)	(0.224)
*N*	6,006	6,003	5,445	5,439

Unstandardized coefficients from Probit regressions are reported with robust standard errors in parentheses. The dependent variable is exercise participation (binary). All models control for age, partnered status, years of education, household income (ln), subjective social status, self-rated health, chronic disease, Hukou, and regional effects. Models 2 and 4 include the self-reported daily hours of unpaid housework. Standard errors are in parentheses. *p < 0.1, **p < 0.05, ***p < 0.01.

The results show clear gender differences. Among male respondents, neither digital work nor unpaid housework is significantly associated with exercise participation. In Models 1 and 2, the coefficients for digital work (*β* = −0.011, *p* > 0.1) and unpaid housework (*β* = 0.008, *p* > 0.1) are both statistically insignificant. This suggests that, within the male subsample, these two forms of time-related engagement are not significantly linked to exercise participation after adjusting for observed covariates.

By contrast, among female respondents, both digital work and unpaid housework are negatively associated with exercise participation. In the baseline female model without unpaid housework (Model 3), digital work is negatively and significantly associated with exercise participation (*β* = −0.177, *p* < 0.01). After unpaid housework is added in Model 4, housework itself is also negatively and significantly associated with exercise participation (*β* = −0.034, *p* < 0.01). Meanwhile, the coefficient for digital work remains negative and statistically significant (*β* = −0.181, *p* < 0.01).

The stability of the digital work coefficient after the inclusion of unpaid housework suggests that the negative association between digital work and women’s exercise participation is not fully accounted for by measured housework time. Instead, the results are consistent with the possibility that work-related digital engagement and unpaid domestic labor operate as two distinct constraints on women’s exercise participation. This pattern provides empirical support for using “Parallel Double Deprivation” as an interpretive concept: women may face overlapping time-related constraints from both work-related digital engagement and domestic labor responsibilities.

However, this finding should be interpreted cautiously. The analysis does not directly measure actual online working time, nor does it provide a formal causal test of mediation. Therefore, the results should be understood as evidence of parallel statistical associations rather than definitive proof of a structural mechanism. Nevertheless, the pattern highlights the importance of considering gendered time constraints when examining the relationship between digital engagement and health-related behavior.

### 3.7 Robustness check: sensitivity to unobservable selection Bias

To assess whether the main associations for digital learning and digital work are sensitive to potential unobservable selection, we performed Oster’s (38) coefficient stability analysis (Table 7). This analysis should be interpreted as a sensitivity test rather than as definitive evidence of causal identification.

**Table 7:** Oster’s bounding analysis for unobservable selection bias.

Variable	Uncontrolled effect	Controlled effect	Oster’s β*	Identified set	Critical δ* for *β* = 0
Digital learning	1.900	1.304	0.813	[0.813, 1.304]	1.798
Digital work	0.610	−0.129	−0.460	[−0.460, −0.129]	−0.428

The controlled effect corresponds to the coefficients from the fully specified baseline regressions. Following Oster (38), *R*_max_ is set to 0.185 (calculated as 1.3 × 0.142, where 0.142 is the *R*^2^ of the controlled model). Oster’s β* is estimated assuming proportional selection (*δ* = 1). The identified set is bounded by the controlled effect and Oster’s β*.

For digital learning, assuming that unobservables are as important as the included observables (*δ* = 1) and setting the maximum attainable *R*^2^ to *R*_max_ = 0.185 (based on 1.3 times the *R*^2^ of the fully controlled model), the adjusted coefficient (β* = 0.813) remains positive. The identified set [0.813, 1.304] does not include zero. Furthermore, the critical δ* is 1.798, suggesting that selection on unobservables would need to be approximately 1.8 times as strong as selection on the observed controls to reduce the estimated association to zero. This result indicates that the positive association between digital learning and exercise participation is relatively robust to proportional selection on unobservables.

For digital work, the sensitivity analysis also supports the stability of the negative association observed in the fully controlled model. The inclusion of covariates changes the uncontrolled positive coefficient (0.610) to a negative coefficient (−0.129), indicating that observed covariates account for an important part of the initial positive association. Oster’s adjusted estimate further shifts the coefficient in the negative direction (β* = −0.460). The identified set [−0.460, −0.129] remains entirely below zero, which suggests that the negative association is unlikely to be fully explained away under the proportional selection assumption. The critical δ* is negative (−0.428), indicating that unobserved selection would need to operate in the opposite direction to the observed selection pattern to move the coefficient to zero. Taken together, these results suggest that the main associations for digital learning and digital work are not highly sensitive to unobservable selection, although they should still be interpreted as robustness evidence rather than causal proof.

## 4 Discussion

This study examined the association between digital connectivity and physical exercise by shifting the analytical focus from the first-level digital divide, defined by access, to the second-level digital divide, defined by functional heterogeneity in internet use. Rather than treating internet use as a uniform exposure, the analysis distinguished among learning, socializing, consumption, entertainment, and work-related digital engagement. The findings offer three main implications for understanding digital engagement and health-related behavior, while remaining subject to the limitations of cross-sectional observational data.

### 4.1 Beyond the binary: functional heterogeneity in digital engagement

First, the findings challenge a binary understanding of internet use by showing that different forms of digital engagement are associated with physical exercise in different directions and magnitudes. While the baseline models (Table 3) indicate a positive association between general internet access and physical exercise, the joint model of functional heterogeneity (Table 4) reveals a more differentiated pattern. In particular, digital learning is positively associated with exercise participation, whereas digital work is negatively associated with exercise participation after other forms of digital engagement are included in the same model.

This pattern is consistent with, but does not conclusively demonstrate, a time-displacement interpretation. Digital learning may be linked to exercise participation through greater exposure to health information, self-improvement resources, or fitness-related knowledge. By contrast, work-related digital engagement may be associated with reduced opportunities for leisure-time physical activity, especially when digital work is connected to extended availability or blurred work–life boundaries. However, because the digital work measure captures work-related reliance on the internet rather than actual time spent working online, this interpretation should be treated as suggestive rather than definitive.

The negative coefficient for digital work in the joint model also resonates with prior discussions of the “autonomy paradox” in digitally mediated work (7). Although telecommuting and digital work tools may increase flexibility in some contexts (39), they may also extend work demands beyond conventional workspaces and working hours (9, 21). In this sense, the present findings add empirical nuance to the literature by suggesting that work-related digital engagement may be less compatible with health-promoting leisure than other forms of internet use. Nevertheless, the magnitude of the digital work association is more modest than that of digital learning, and the results should not be interpreted as evidence that digital work uniformly or causally suppresses physical exercise.

### 4.2 Digital entertainment and rural exercise participation

Second, the hukou-stratified results suggest that the positive association between digital entertainment and exercise participation is more evident among rural respondents than among urban respondents (Table 5). This finding complicates the common assumption that entertainment-oriented screen use is necessarily sedentary or health-eroding. In the present sample, entertainment-oriented digital engagement is positively associated with exercise participation in rural areas, while no significant association is observed among urban respondents.

One possible explanation is that digital entertainment platforms may have different social meanings and behavioral implications across spatial contexts. In rural settings, where formal fitness facilities and organized leisure opportunities may be less available (40–42), entertainment platforms—especially short-video and social media applications—may expose users to exercise-related content or facilitate informal collective activities. For example, platform-based content may be connected to locally embedded activities such as square dancing or other group-based exercise practices. This interpretation is broadly consistent with research suggesting that digital platforms may support social capital formation and offline participation under certain conditions (12, 24, 43–45).

However, this interpretation should remain cautious. The present analysis does not directly measure exposure to fitness-related content, participation in platform-organized activities, or the availability of local exercise infrastructure. Therefore, the rural association should be understood as evidence of spatial heterogeneity in the digital–exercise relationship, rather than direct proof that digital entertainment causes rural exercise participation.

### 4.3 Parallel double deprivation as an interpretive lens

Third, the gender-stratified and mechanism analyses provide tentative support for using “Parallel Double Deprivation” as an interpretive lens for understanding women’s exercise participation in the digital era. The results show that, among women, both digital work and unpaid housework are negatively associated with exercise participation, whereas neither association is statistically significant among men (Table 6). Moreover, the negative association between digital work and women’s exercise participation remains stable after unpaid housework is added to the model.

These findings do not prove a causal mechanism, but they suggest that work-related digital engagement and unpaid domestic labor may represent two distinct time-related constraints for women. This pattern extends the literature on the “second shift” by indicating that domestic labor remains relevant for health-related leisure even in a highly digitalized context (27, 30, 31). At the same time, the findings suggest that digital work may constitute an additional layer of constraint, particularly when work-related connectivity increases the permeability of boundaries between paid work and private life (7, 9).

The concept of “Parallel Double Deprivation” is therefore best understood as a cautious theoretical interpretation rather than a fully validated mechanism. It does not imply that digital work and unpaid housework always operate in the same way, nor that their relationship with exercise participation is necessarily causal. Rather, the concept highlights the possibility that women’s leisure-time health behavior may be shaped by multiple, coexisting demands on time. This interpretation is consistent with gender and time-use research showing that women often face greater responsibility for unpaid domestic labor and may experience flexible or digital work arrangements differently from men (26, 28, 31–33). Future longitudinal and time-diary studies are needed to test whether these parallel constraints operate through actual time displacement, psychological fatigue, or other pathways.

## 5 Conclusion

Using a nationally representative sample from the CFPS 2022, this study examined the association between digital engagement and physical exercise participation in China. The findings suggest that internet access alone does not fully capture the relationship between digitalization and health-related behavior. Rather, different forms of digital engagement are associated with physical exercise in different ways. Digital learning is positively associated with exercise participation, whereas digital work is negatively associated with exercise participation in the fully adjusted joint model. These findings indicate that the second-level digital divide, reflected in the functional heterogeneity of internet use, is important for understanding digital health inequalities.

The study also identifies meaningful subgroup differences. Digital entertainment is positively associated with exercise participation among rural respondents, suggesting that entertainment-oriented digital platforms may have different behavioral implications across spatial contexts. In addition, among women, both digital work and unpaid housework are negatively associated with exercise participation. This pattern provides tentative support for interpreting women’s exercise participation through the lens of “Parallel Double Deprivation,” understood as the coexistence of work-related digital engagement and unpaid domestic labor as potential time-related constraints. However, given the cross-sectional design and the indirect measurement of digital work, these results should be interpreted as evidence of statistical associations rather than definitive causal mechanisms.

### 5.1 Policy implications

These findings have several implications for public health promotion and labor-related policy discussions in the digital era.

#### 5.1.1 Work–life boundaries and digital labor

The negative association between digital work and exercise participation suggests that work-related digital engagement may be relevant to debates on work–life boundaries and employee well-being. Policymakers and organizations may consider measures that reduce excessive after-hours connectivity and protect non-work time, including clearer expectations regarding digital availability outside regular working hours. Although the present study cannot directly evaluate the causal effects of a formal “Right to Disconnect,” the findings suggest that such policy debates may have public health relevance in addition to labor-rights significance.

#### 5.1.2 Gender-responsive health interventions

The gendered pattern observed in the analysis suggests that generic exercise-promotion campaigns may be insufficient if they overlook unequal time constraints. Public health interventions should pay closer attention to the distribution of unpaid domestic labor and the ways in which flexible or digitally mediated work may be experienced differently by women and men. Programs designed to promote physical activity among women may therefore need to address not only individual motivation but also the structural and household-level constraints that limit leisure-time exercise.

#### 5.1.3 Leveraging digital platforms for rural health promotion

The positive association between digital entertainment and exercise participation among rural respondents suggests that digital platforms may offer opportunities for localized health promotion in rural areas. Public health agencies and community organizations could consider using short-video and social media platforms to disseminate culturally appropriate and locally relevant exercise content. Such initiatives should be designed carefully to avoid assuming that digital entertainment is uniformly beneficial, but they may provide a low-cost channel for reaching communities with more limited access to formal fitness infrastructure.

### 5.2 Limitations and future research

This study has several limitations. First, although Oster’s coefficient stability analysis was used to assess sensitivity to unobservable selection, the cross-sectional nature of the CFPS 2022 data limits causal inference. Future research should employ longitudinal designs to examine how digital engagement and physical exercise change over time.

Second, the measures of exercise and digital engagement are self-reported, which may introduce recall bias and social desirability bias. In addition, the digital work indicator captures perceived work-related reliance on the internet rather than actual time spent working online. Future studies could integrate objective measures, such as wearable-device data, smartphone screen-time logs, or time-diary data, to provide a more precise assessment of time allocation and potential displacement processes.

Third, the “Parallel Double Deprivation” concept is supported in this study as an interpretive framework rather than as a fully established causal mechanism. Future research should test this framework using longitudinal, mediation, and mixed-method designs. Qualitative studies would also be valuable for examining how women experience the combined pressures of work-related digital connectivity and unpaid domestic labor in everyday life.

## Data Availability

Publicly available datasets were analyzed in this study. This data can be found at: “The datasets analyzed for this study can be found in the China Family Panel Studies (CFPS) repository, administered by the Institute of Social Science Survey (ISSS) at Peking University. The data are publicly available upon request at http://www.isss.pku.edu.cn/cfps/index.htm”.
