# Supplementary material for: The “parallel double deprivation”: how functional digital divides and gendered time constraints shape physical exercise participation in China
Source: Front Public Health. 2026 Jul 8;14:1841526. doi: 10.3389/fpubh.2026.1841526 (PMC13388787; doi:10.3389/fpubh.2026.1841526)
Supplement: Supplementary file 1 [file Table_1.doc]

**TABLE S1 Variance Inflation Factors for the Joint Model**

| **Variable** | **VIF** | **1/VIF** |
| --- | --- | --- |
| Digital learning | 1.45 | 0.688 |
| Digital socializing | 1.24 | 0.807 |
| Digital consumption | 1.47 | 0.680 |
| Digital entertainment | 1.29 | 0.773 |
| Digital work | 1.42 | 0.703 |
| Age | 1.97 | 0.506 |
| Gender (male) | 1.05 | 0.951 |
| Partnered (Yes) | 1.52 | 0.657 |
| Years of education | 1.29 | 0.773 |
| Ln_household income | 1.22 | 0.823 |
| Subjective social status | 1.10 | 0.907 |
| Self-rated health | 1.21 | 0.830 |
| Chronic disease (Yes) | 1.15 | 0.872 |
| Urban (Yes) | 1.17 | 0.854 |
| Region |  |  |
| West | - | - |
| Central | 1.52 | 0.657 |
| East | 1.60 | 0.626 |
| **Mean VIF** | **1.36** |  |

VIFs were calculated using an auxiliary OLS regression with the same set of predictors as the joint Probit model. The results are based on the first imputed dataset. All VIF values were below the conventional threshold of 5, suggesting that multicollinearity was not a serious concern.
